# The Prevention of Shock during Operations

**Published:** 1901-10-26

**Authors:** 


### THE PREVENTION OF SHOCK DURING OPERATIONS.

The first effect of the introduction of anæsthetics was to facilitate the performance of the operations which were then in use. The next was to render possible many operations which before had not been dreamed of. So with antiseptics, their first effect was seen in increased safety, but this was quickly followed by a vast extension of surgical proceedings, and now the time appears to have arrived when the real obstruction to further progress is to be found in many cases in the shock of the operation. If patients could but live through the operation and escape the collapse of the first few hours, still further advances could be made even upon the present position of surgery. The treatment for shock which is now almost universally practised is the intravenous injection of saline solution, and no one who has witnessed its marvellous efficacy in restoring apparently hopeless cases can hesitate as to its efficacy and utility. As commonly employed, however, as a remedy rather than as a preventative, this operation is often performed under very disadvantageous circumstances. The occasion for it is a grave emergency, the instrument may not be at hand, the fluid employed may not be above suspicion, and thus accidents may happen. Mr. George Brown,[1] of Leeds, now urges that there are cases in which this method of transfusion should not be reserved for use in emergencies, but should be undertaken as a part of the major operation itself, a special surgeon, with proper apparatus and carefully prepared solution, being employed for this purpose alone. He says that in several cases he has operated under such conditions. So soon as the patient is under ether a competent surgeon opens a selected vein and begins to transfuse.

As the major operation proceeds so *pari passu* does the intravenous injection, the amount injected being governed by the state of the pulse and the loss of blood consequent on the removal of the tumour. Speaking of the removal of myomata, where much blood has been lost before the operation, Mr. Brown says that as a rule he has used about five pints of fluid. We think that there is much common sense in what Mr. Brown says. It is quite clear that transfusion, done by a special assistant engaged for that office alone, and done with proper apparatus and with full preparation, stands on a very different footing from the same proceeding hurriedly called for, only when the failing pulse and the dilated pupil foreshadow catastrophe on the table. In great operations in the future, then, we may expect to see the operator arrive accompanied not only by "the gentleman with the nose-bag," as we have heard the anæsthetist described, but by another important personage, the transfuser with all his special apparatus. Thus we see prospects of the birth of a new specialty.

[1] Brit. Med. Jour., Oct. 19.

